# LINE‐1 hypomethylation characterizes the inflammatory response in coeliac disease associated‐intestinal mucosa and small bowel adenocarcinomas

**DOI:** 10.1002/path.6371

**Published:** 2024-11-27

**Authors:** Laura Libera, Alessandro Vanoli, Nora Sahnane, Muhammad Adnan, Camilla Guerini, Giovanni Arpa, Paola Ilaria Bianchi, Marco Vincenzo Lenti, Gino Roberto Corazza, Stefano La Rosa, Antonio Di Sabatino, Daniela Furlan

**Affiliations:** ^1^ Unit of Pathology, Department of Medicine and Technological Innovation University of Insubria Varese Italy; ^2^ Hereditary Cancer Research Centre, Department of Medicine and Technological Innovation University of Insubria Varese Italy; ^3^ Department of Molecular Medicine, Unit of Anatomic Pathology University of Pavia Pavia Italy; ^4^ Unit of Anatomic Pathology Fondazione IRCCS San Matteo Hospital Pavia Italy; ^5^ Unit of Anatomic Pathology Azienda Socio Sanitaria Territoriale (ASST) dei Sette Laghi Varese Italy; ^6^ First Department of Internal Medicine Fondazione IRCCS San Matteo Hospital Pavia Italy; ^7^ Department of Internal Medicine and Medical Therapeutics University of Pavia Pavia Italy

**Keywords:** coeliac disease, immune‐mediated disorders, LINE‐1 hypomethylation, LINE‐1 retrotransposition, small bowel adenocarcinomas

## Abstract

Long interspersed nuclear elements 1 (LINE‐1) are the most abundant and the only autonomous mobile elements in the human genome. When their epigenetic repression is removed, it can lead to disease, such as autoimmune diseases and cancer. Coeliac disease (CeD) is an immune‐mediated disease triggered by an abnormal T‐cell response to dietary gluten and a predisposing condition of small bowel adenocarcinoma (SBA), frequently characterized by epigenetic alterations. The aim of this work was to assess LINE‐1 methylation by bisulphite pyrosequencing and NanoString® gene transcription analysis in 38 CeD‐SBAs compared with 25 SBAs associated with Crohn's disease (CrD‐SBAs) and 25 sporadic SBAs (S‐SBA). Both analyses were also performed in duodenal mucosae from 12 untreated CeD patients (UCD) and 19 treated CeD patients (TCD), and in 11 samples of normal intestinal mucosa to better investigate the role of LINE‐1 deregulation in CeD and in CeD‐SBA. A significant loss of LINE‐1 methylation was observed in CeD‐SBAs and in mucosae from UCD patients (with very similar methylation levels) compared with controls. By contrast, a restoration of normal LINE‐1 methylation levels was found in TCD mucosae after a strict gluten‐free diet. LINE‐1 hypomethylation does not lead to expression of ORF1 and ORF2, with the only exception being for one CeD‐SBA. The expression analysis of enzymes modulating DNA methylation and inflammatory genes confirmed that CeD‐SBA shared a very similar expression profile of UCD mucosae showing a strong upregulation of genes involved in inflammation, immune response, and T‐cell activity compared with TCD mucosae. For the first time, this work demonstrates that loss of DNA methylation is an intrinsic epigenetic feature of CeD, accompanying the immune response as a reversible mechanism in patients following a strict gluten‐free diet, and suggests the possible role of LINE‐1 hypomethylation in promoting cell adaptability during the gliadin‐related inflammatory process. © 2024 The Author(s). *The Journal of Pathology* published by John Wiley & Sons Ltd on behalf of The Pathological Society of Great Britain and Ireland.

## Introduction

Long interspersed nuclear elements 1 (LINE‐1) are the most abundant class of retrotransposons in mammals and account for 17% of the human genome. They are the only autonomous and active mobile DNAs in the human genome, using RNA intermediates via retrotransposition [1]. Activation of full‐length LINE‐1 plays a crucial role in promoting adaptability, stress response, and the immune system with the production of type I interferons (IFN‐I) [2, 3, 4]. They contain a 5’‐untranslated region (5’‐UTR) with an antisense promoter [5], two open reading frames (ORF1 and ORF2) required for LINE‐1 retrotransposition, and a 3’‐UTR region with a poly‐A tract [6, 7].

Regulation of LINE‐1 is critical to maintain genome integrity and they are normally suppressed in adult tissues via histone deacetylation, H3K9me3, and DNA methylation [8, 9, 10, 11]. When this epigenetic repression is out of control, it can lead to disease, such as cancer, autoimmune disease, and metabolic and neurological disorders [12].

Many recent studies from large cancer genome consortia have demonstrated that LINE‐1 retrotransposons are widely activated in many different types of human cancers [13, 14, 15, 16] and that their dysregulation may contribute to cancer transformation and progression. Moreover, hypomethylated and highly expressed LINE‐1 have been demonstrated in autoimmune diseases [17, 18, 19], and several cell studies confirmed that LINE‐1 retrotransposition activates the production of IFNβ by RNA sensors, inducing an immune response [20, 21].

Coeliac disease (CeD) is an immune‐mediated disease triggered by an abnormal T‐cell response to dietary gluten in genetically susceptible individuals. CeD is a predisposing condition of small intestinal adenocarcinoma (SBA) and enteropathy‐associated T‐cell lymphoma, and the mortality from lymphoproliferative diseases in CeD patients has been reported to increase significantly 2–5 years after a CeD diagnosis [22, 23]. A recent methylome study performed in duodenal mucosa samples from CeD patients described two distinct methylation profiles characterized by a remarkable loss of CpG island boundaries [24], and genome‐wide association studies mapped CeD susceptibility genetic variants at or near to the hotspot regions known for epigenetic modifications [25]. In addition, it is well established that small bowel adenocarcinomas associated with CeD (CeD‐SBAs) are mainly characterized by epigenetic alterations such as *MLH1* methylation leading to microsatellite instability (MSI) in comparison to controls (i.e. sporadic SBAs, S‐SBAs) and SBAs associated with Crohn's disease (CrD‐SBAs) [26], although the molecular mechanisms underlying these alterations remain unclear.

For the first time in this work, we assessed LINE‐1 methylation levels in a large series of SBAs including CeD‐SBAs, S‐SBAs, and CrD‐SBAs, and we evaluated the hypothesis that LINE‐1 hypomethylation may be a distinctive feature not only of CeD‐SBA but also of the small intestinal mucosa of patients with active CeD, leading or not to the activation of the retrotransposons during the inflammatory response.

## Materials and methods

### Case series presentation

This retrospective study included 88 patients with pathologically confirmed primary non‐familial, non‐ampullary SBA associated with either CeD (CeD‐SBA, *n* = 38), Crohn's disease (CrD‐SBA, *n* = 25), or without any known predisposing conditions (i.e. sporadic, S‐SBA, *n* = 25), who had surgical resection and completed survival data between 1995 and 2020 from 28 tertiary referral Italian CeD centres, Inflammatory bowel disease centres, Oncology centres, Abdominal Surgery Units, and Pathology Anatomy Units participating in the Small Bowel Cancer Italian Consortium. Histological evaluations, including mismatch repair (MMR) protein system immunohistochemical status, MSI, and *MLH1* promoter methylation, were performed for all SBA cases according to the protocols previously described by Vanoli *et al* [26].

Moreover, duodenal mucosae from 12 untreated (UCD) and 19 treated CeD patients (TCD) were collected and analysed, as well as from 11 control subjects with normal intestinal mucosa, in whom CeD, CrD, and SBA were excluded. Also, for UCD patients, a CeD diagnosis was based on serum IgA anti‐endomysial and/or anti‐tissue transglutaminase antibody positivity associated with typical duodenal histopathological lesions (Corazza–Villanacci grade B2). In all the TCD patients, a good histological response was demonstrated after at least 12 months of a strict gluten‐free diet (GFD), showing Corazza–Villanacci grade A.

This study was approved by the Ethics Committee of Pavia (protocol number 20140003980).

### DNA extraction and LINE‐1 methylation analysis

DNA was extracted from three representative 8‐μm‐thick sections obtained from 123 formalin‐fixed and paraffin‐embedded (FFPE) samples using the Maxwell® DNA FFPE Kit and Maxwell 16 system (Promega, Madison, WI, USA) according to the manufacturer's protocol and quantified using the Qubit dsDNA High Sensitivity Assay Kit (Invitrogen, Thermo Fisher Scientific Inc., Waltham, MA, USA). The methylation status of global LINE‐1 (GenBank accession number M80343.1) was evaluated by bisulphite‐PCR and pyrosequencing. Bisulphite modification of genomic DNA (100–300 ng) was performed using the EZ DNA Methylation Kit (Zymo Research, Irvine, CA, USA) according to the manufacturer's recommendations. Bisulphite‐modified DNA was amplified and sequenced, addressing four CpG sites by using LINE‐1 primers and the protocol previously reported by Stefanoli *et al* [27]. Human methylated and non‐methylated (WGA) DNA sets (Zymo Research) were used as positive and negative controls in each experiment. The cut‐off value for LINE‐1 methylation test was set at 60% to discriminate hypomethylated from hypermethylated cases, accordingly to our previous publications [28, 29].

### RNA extraction and NanoString® expression analysis

Expression analysis was performed on 10 CeD‐SBAs, 10 S‐SBAs, and 22 CeD mucosae (18 TCD and 4 UCD) and conducted using the NanoString® nCounter® gene expression platform (NanoString Technologies, Seattle, WA, USA) using a custom gene panel which included the following 28 genes involved in the regulation of DNA methylation and in specific inflammatory pathways associated with CeD and CrD: *CD3D* (NM_000732.4), *CD3E* (NM_000733.2), *CD3G* (NM_000073.2), *CD4* (NM_000616.4), *CD8A* (NM_001768.5), *CD8B* (NM_172099.2), *DNMT1* (NM_001379.2), *DNMT3A* (NM_022552.4), *DNMT3B* (NM_175850.1), *INFA1* (NM_024013.1), *INFB1* (NM_002176.2), *INFG* (NM_000619.2), *IL12A* (NM_000882.2), *IL12B* (NM_002187.2), *IL15* (NM_172174.1), *IL18* (NM_001562.2), *IL1B* (NM_000576.2), *IL2* (NM_000586.2), *IL21* (NM_021803.2), *IL23A* (NM_016584.2), *IL6* (NM_000600.3), *LINE1‐ORF1* (M19503.1:1706), *LINE1‐ORF2* (M19503.1:2700), *RB1* (NM_000321.1), *TET1* (NM_030625.2), *TET2* (NM_001127208.2), *TET3* (NM_001287491.2), and *TNFA* (NM_000594.2), as shown in supplementary material, Table S1. Six reference genes were included for gene expression normalization (*TBC1D10B*, NM_015527.3; *TBP*, NM_001172085.1; *TFRC*, NM_003234.1; *TLK2*, XM_011524223.1; *TMUB2*, NM_024107.2; and *UBB*, NM_018955.2), while the *MLH1* gene (NM_000249.2) was included as a control gene to validate the NanoString® results, as data on the immunohistochemical expression of MLH1 protein and the *MLH1* methylation status were available for all the SBAs tested with the NanoString® custom panel [26, 30].

Briefly, RNA was obtained for each sample as previously described by Bolzacchini *et al* [31] by using the Maxwell® RNA FFPE Kit and Maxwell 16 system (Promega). About 100–300 ng (24 samples) or 42–99 ng (18 samples) of total RNA was hybridized overnight with a 3’‐biotinylated capture probe and a 5’‐reporter probe from the custom panel code set following the manufacturer's recommendations. Hybridized samples were run on the NanoString nCounter preparation station using the high‐sensitivity protocol and scanned at a high scan resolution (280 FOVs, fields of view) on the nCounter Digital Analyzer.

Raw data for each sample and gene were normalized to internal controls to eliminate technical variability of the assay, and then counts were normalized to the geometric mean of six endogenous housekeeping genes followed by log_2_ transformation by using NanoString nSolver™ Analysis Software. Normalized log_2_ gene counts were compared with molecular and immunostaining features.

To compare the expression profiles of the different subsets of cases, log_2_ fold‐change and *p* value by Student's *t*‐test were calculated for each gene.

### Statistical analysis of clinicopathological data

Statistical analysis was performed using Student's *t*‐test, ANOVA followed by the Bonferroni test, and Pearson's *χ* ^2^ test. The Kaplan–Meier method was used to estimate the overall survival probability based on log‐rank test. *p* < 0.05 was considered significant. The Stata Statistical Software release 17 (StataCorp LLC, College Station, TX, USA) and GraphPad v.5.0 software (GraphPad Software Inc., San Diego, CA, USA) were used for the statistical analyses.

## Results

### Clinicopathological characteristics of the series

Clinicopathological data of the SBA patients are summarized in Table 1. The median age at the diagnosis of SBA was 60.50 (range 52–68) years; the median age of SBA onset in the group of S‐SBAs was 65 (range 62–73) years, while in the CeD‐SBA group it was 54 (range 42–65) years and in the CrD‐SBA group it was 59 (range 54–69) years. The male‐to‐female ratio was 2.13 (17:8) in the S‐SBA group, 1 (19:19) in the CeD‐related group, and 1.78 (16:9) in the CrD‐related group. The median follow‐up after the diagnosis of SBA was 44 (range 19–72) months for S‐SBAs, 40 (range 2–94) months for CeD‐SBAs, and 28 (range 5–72) months for CrD‐SBAs. In this period, only four (10.5%) CeD‐SBA patients died of cancer disease compared with 13 (52%) and 17 (69%) patients of the S‐SBA and CrD‐SBA groups, respectively. The median time between CeD and CeD‐SBA diagnosis was 2 years, and only one patient affected by CeD‐SBA showed type 1 refractory CeD. Histologically, most cancers were classified as SBA‐not otherwise specified. Twenty‐one out of 38 CeD‐SBAs (55%), 4 out of 25 (16%) CrD‐SBAs, and 4 out of 25 (16%) S‐SBAs showed MSI.

**Table 1:** Clinicopathological and immunohistochemical features of the 88 small bowel adenocarcinomas.

		*n* cases (%)
Age at SBA diagnosis (25th–75th percentile)		Median 60.50 (range 52–68) years
Female gender		36 (37%)
Aetiology	Crohn's disease	25 (28%)
	Coeliac disease	38 (44%)
	Sporadic	25 (28%)
Tumour site	Duodenum	11 (13%)
	Jejunum	45 (51%)
	Ileum	32 (36%)
AJCC stage	I	5 (6%)
	II	46 (52%)
	III	27 (31%)
	IV	10 (11%)
Histological subtype	SBAs‐NOS	59 (67%)
	Medullary SBAs	8 (9%)
	PCCs	7 (8%)
	Mixed‐PCG‐SBAs	14 (16%)
MMR deficiency		29 (33%)

AJCC, American Joint Committee on Cancer; MMR, mismatch repair; mixed‐PCG‐SBA, mixed‐poorly cohesive‐glandular small bowel adenocarcinoma; PCC, poorly cohesive carcinoma; SBA, small bowel adenocarcinoma; SBA‐NOS, small bowel adenocarcinoma, not otherwise specified.

Concerning the duodenal mucosae, the median patient age was 34 (range 30–42) years for UCD patients, 34 (range 26–46) years for TCD patients, and 63 (range 57–71) years for controls. The male‐to‐female ratio was 0.09 (1:11) for the UCD group, 0.46 (6:13) for the TCD group, and 2.67 (8:3) for the control group.

### CeD‐SBAs are characterized by LINE‐1 hypomethylation

**Figure 1:**
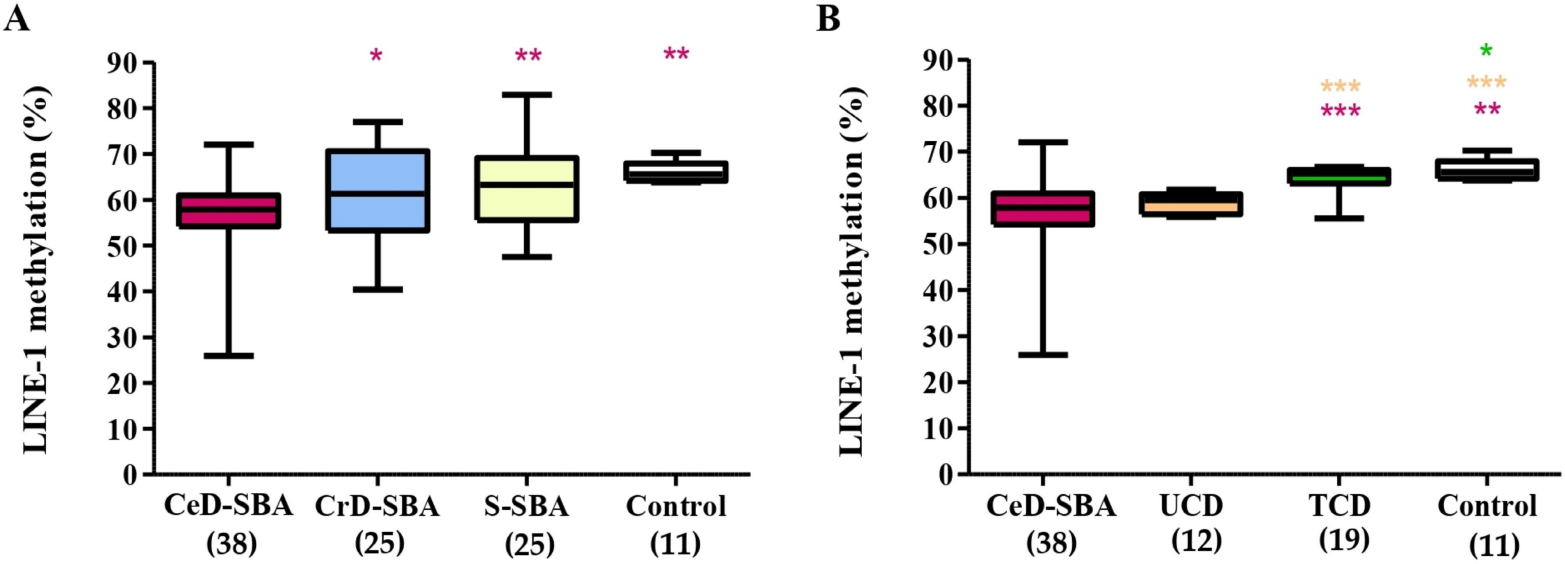
Distribution of LINE‐1 methylation in small bowel adenocarcinomas (SBAs) and mucosae. (A) Box‐plot results of LINE‐1 methylation analysis among coeliac disease SBA (CeD‐SBA, *n* = 38, purple box), Crohn's disease SBA (CrD‐SBA, *n* = 25, blue box), sporadic SBA (S‐SBA, *n* = 25, yellow box), and normal mucosae from controls (Control, *n* = 11, white box). (B) Box‐plot results of LINE‐1 methylation analysis among coeliac disease SBA (CeD‐SBA, *n* = 38, purple box), duodenal mucosae from untreated CeD patients (UCD, *n* = 12, orange box) and treated CeD patients (TCD, *n* = 19, green box), and normal mucosae from controls (Control, *n* = 11, white box). Asterisks indicate the level of statistical significance: *p = 0.05–0.005, **p = 0.005–0.0001, ***p < 0.0001. The colour of the asterisks indicates the groups of comparison.

Quantitative LINE‐1 methylation analysis was possible for all 88 SBAs (38 CeD‐SBAs, 25 CrD‐SBAs, and 25 S‐SBAs) and 11 control small bowel mucosae for comparison. In CeD‐SBAs, the distribution of LINE‐1 methylation levels varied from 25.9% to 72% (average 55.3%); in CrD‐SBAs, from 40.4% to 77% (average 61.08%); in S‐SBAs, from 47.5% to 82.93% (average 63.72%); and in control mucosae, the LINE‐1 methylation rate ranged from 63.75% to 70.25% (average 66.21%). Comparing these results, the CeD‐SBA group showed a significantly lower level of LINE‐1 methylation compared with the CrD‐SBAs (*p* = 0.0349), the S‐SBAs (*p* = 0.0021), and the control small bowel mucosae (*p* = 0.0013, Figure 1A).

When a cut‐off of 60% was considered for LINE‐1 methylation to discriminate hypomethylated (hypo‐SBA) from hypermethylated (hyper‐SBA) cases, CeD‐SBAs were enriched by hypomethylated cases (66%, 25/38 CeD‐SBAs) compared with CrD‐SBAs (40%, 10/25 CrD‐SBAs, *p* = 0.0692, trend) and S‐SBAs (32%, 8/25 S‐SBAs, *p* = 0.0110). This result suggested that a lower level of global methylation characterizes CeD‐SBAs, regardless of MSI status and *MLH1* methylation. Indeed, both MSI CeD‐SBAs (13 out of 21 MSI CeD‐SBAs) and MSS CeD‐SBAs (12 out of 17 MSS CeD‐SBAs) showed LINE‐1 hypomethylation. Interestingly, the co‐occurrence of LINE‐1 hypomethylation and *MLH1* hypermethylation was significantly associated with CeD‐SBAs (34%, 13/38 CeD‐SBAs) compared with CrD‐SBAs (4%, 1/25 CrD‐SBAs) and S‐SBAs (0%, 0/25 S‐SBAs, *χ* ^2^ *p* value = 0.0002).

Interestingly, no difference in the overall survival analysis was observed when LINE‐1 hypo‐ and hyper‐SBAs were compared (supplementary material, Figure S1A), even when the three groups of SBAs were separated. As previously reported, CeD‐SBA patients showed a more favourable outcome compared with CrD‐SBA and S‐SBA patients (supplementary material, Figure S1B), likely due to the immune signatures of CeD‐SBAs [26, 32].

### Restoration of normal LINE‐1 methylation levels in TCD mucosae

To better understand whether the hypomethylation pattern is distinctive of cancer or is an intrinsic feature of CeD mucosae, the methylation analysis was extended to 12 duodenal mucosae from UCD patients (Corazza–Villanacci grade B2) and 19 duodenal mucosae from TCD patients who followed a strict GFD with histologic response (Corazza–Villanacci grade A).

As shown in Figure 1B, we compared the LINE‐1 methylation levels of the CeD‐SBA, UCD, and TCD groups with those of control normal mucosae. The percentage of LINE‐1 methylation gradually decreased from control mucosae (66.2%) to CeD‐SBAs (55.3%), passing through TCD (64.2%) and UCD (58.8%). Remarkably, although no significant difference was observed between the CeD‐SBA and UCD groups, both showed a significantly lower level of global methylation compared with control mucosae (*p* = 0.0013 and *p* < 0.0001, respectively).

On the contrary, the TCD group had a significantly higher level of LINE‐1 methylation (64.2%) with respect to the CeD‐SBA (*p* = 0.0006) and UCD groups (*p* < 0.0001). This finding suggests that the GFD helps in the restoration of LINE‐1 methylation levels towards the normal condition, even though the TCD group showed a significantly lower level of methylation compared with control mucosae (*p* = 0.0463).

### Expression of enzymes catalysing DNA methylation and inflammatory genes

To better clarify the link between LINE‐1 hypomethylation, inflammation, and CeD, a gene expression analysis of 29 genes involved in DNA methylation and inflammation (including LINE‐1 ORF1 and ORF2) was conducted on 10 CeD‐SBAs (six MSI and four MSS) and compared with the expression profiles of 10 S‐SBAs (two MSI and eight MSS), 4 UCD and 18 TCD mucosae. NanoString® gene expression data are available in supplementary material, Table S2. The NanoString® *MLH1* expression results were compared with MLH1 protein expression and methylation data available for SBAs to validate the custom panel. A complete correspondence between MLH1 protein expression and *MLH1* NanoString® results was observed for all the analysed SBAs (*p* < 0.0001), indicating the good performance of the custom assay (supplementary material, Figure S2).

**Figure 2:**
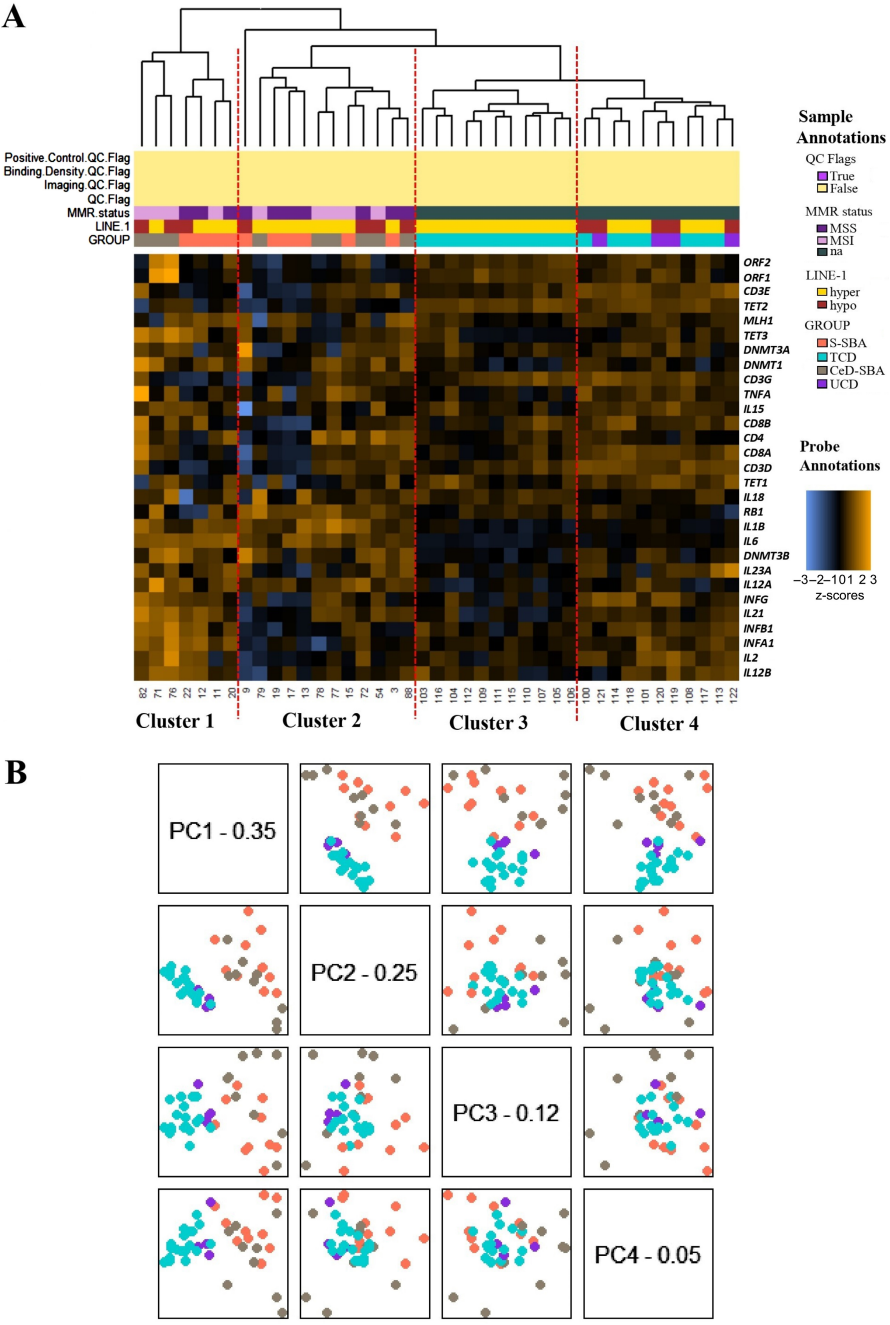
(A) Heatmap of the normalized data, scaled to give all genes equal variance, generated via unsupervised clustering. Expression values are scaled by gene to have a mean of 0 and a standard deviation (SD) of 1 and then truncated at ±3 SDs to preserve greater clarity in colour change within the largest proportion of data (99% of the data should fall within ±3 SDs). Sample annotations are listed at the top of the heatmap: MMR status (MSS in violet; MSI in pink; not available in green); LINE‐1 status (hypermethylated in yellow; hypomethylated in red); class groups (S‐SBA in orange; CeD‐SBA in grey; TCD in light blue; UCD in violet). The genes are displayed in rows. Each column is a unique sample, with a sample label displayed below the heatmap. MMR, mismatch repair status; MSI, presence of microsatellite instability; MSS, absence of microsatellite instability, na, not available; hyper, LINE‐1 hypermethylated; hypo, LINE‐1 hypomethylated; S‐SBA, sporadic SBA, CeD‐SBA, coeliac disease SBA; TCD, mucosae from coeliac disease patients in treatment; UCD, mucosae from coeliac disease untreated patients. (B) Principal component analysis maps high‐dimensional datasets onto a smaller number of highly informative dimensions. The figure shows the first four principal components of the gene expression data plotted against each other and coloured by class groups (S‐SBA in orange; CeD‐SBA in grey; TCD in light blue; UCD in violet). This plot shows the clustering of TCD and UCD mucosae separated from SBA samples.

At first, the unsupervised clustering and the principal component analysis (PCA) identified an outlier case (case 87) characterized by the upregulation of 18 out of 29 target genes (namely, LINE‐1 ORF1 and ORF2, *RB1*, *TET1*, *INFA1*, *INFB1*, *INFG*, *IL1B*, *IL2*, *IL12A*, *IL12B*, *IL15*, *IL21*, *IL23A*, *CD3G*, *CD4*, *CD8B*, and *TNFA*) together with a marked hot profile (supplementary material, Figure S3, highlighted with a red dotted square). Interestingly, this case showed a long cancer‐free period (20 years) from CeD diagnosis to cancer onset, with poor compliance to the GFD. Despite the overexpression of LINE‐1 ORF1 and ORF2 and *TET1*, the outlier showed a mean level of LINE‐1 methylation of 60.73%. As this outlier clustered separately from the tested samples and flattened the heatmap results, a second unsupervised clustering analysis and PCA were performed excluding this case. The results of this second analysis are reported in Figure 2.

As expected, both the heatmap (Figure 2A) and PCA (Figure 2B) showed that CeD mucosae (TCD in light blue and UCD in purple) clustered separately from SBA samples (CeD‐SBA in grey and S‐SBA in orange). In Figure 2A, four clusters can be identified from left to right in the heatmap resulting from the unsupervised clustering analysis. The first cluster grouped together three CeD‐SBAs (cases 82, 71, and 76) and four S‐SBAs (cases 22, 12, 11, and 20), and it is characterized by extended gene upregulation. Cluster 2 is equally composed of six CeD‐SBAs (cases 79, 78, 77, 72, 54, and 88) and six S‐SBAs (case 9, 19, 17, 13, 15, and 3). Cluster 3 is characterized by only TCD samples (*n* = 11) and showed the coldest expression profile among these series of cases, and finally, in cluster 4, all the four UCD (cases 121, 120, 119, and 122) and the remaining seven TCD mucosae are grouped together. The separation between CeD mucosae and SBA is further demonstrated by the PCA (Figure 2B), where the UCD (purple dots) and TCD mucosae (light blue dots) segregate in the lower‐left corner of the principal component 1 graph (PC1), while CeD‐SBA (grey dots) and S‐SBA (orange dots) appear in the upper‐right corner of the PC1 graph. No significant correlation between the clusters and SBA histological subtype was found.

**Figure 3:**
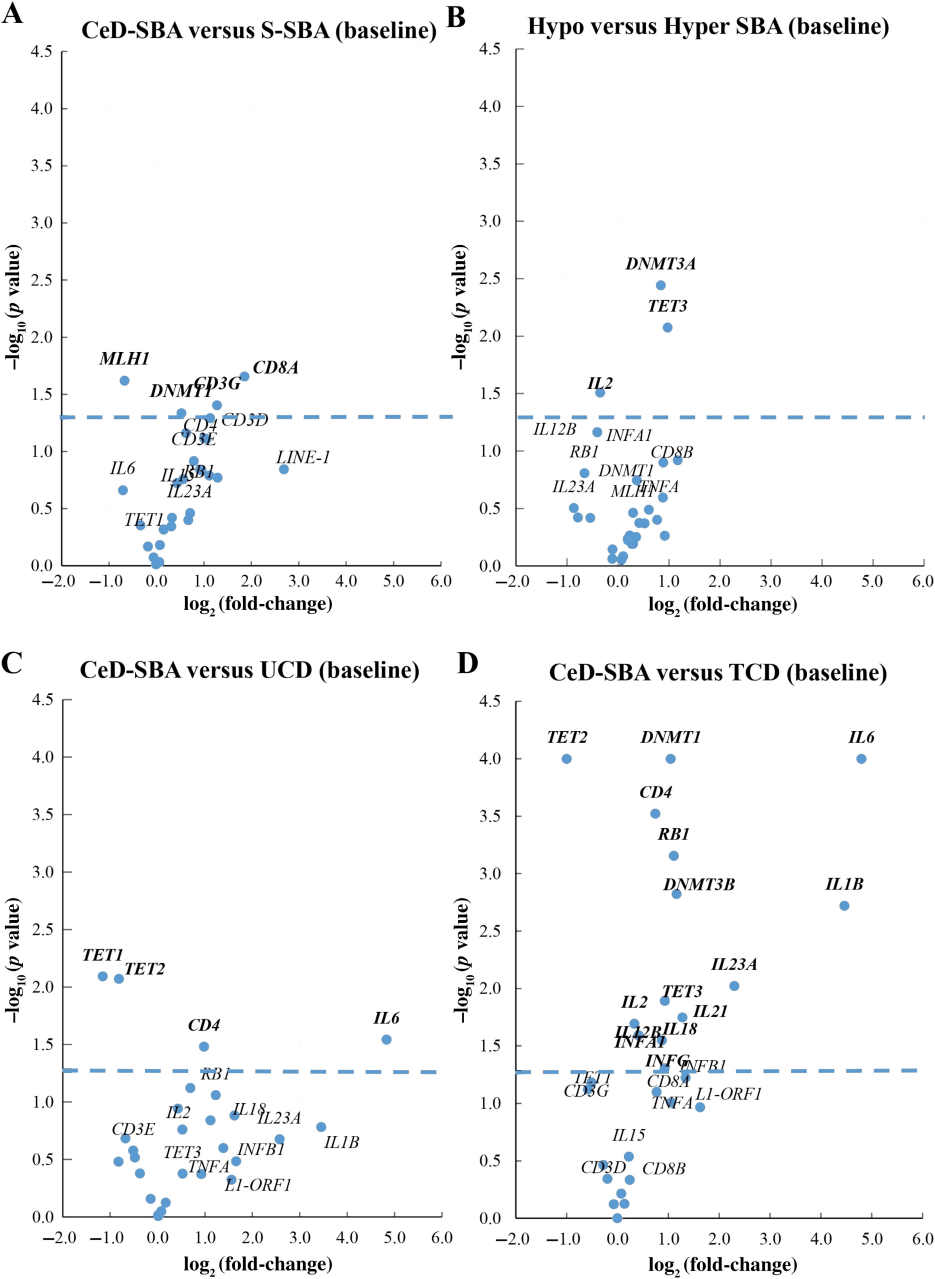
(A–D) Volcano plots displaying each gene's transcript fold‐change (or difference on the log_2_ scale) and significance (*p* value) between CeD‐SBA and S‐SBA (A), between hypomethylated and hypermethylated SBA (B), between CeD‐SBA and UCD (C), and between CeD‐SBA and TCD (D) represented along the *x*‐axis, with the significance (*p* value) along the *y*‐axis. Genes that have greater statistical significance appear higher on the plot. Genes that have greater differential expression versus the baseline group (S‐SBA, hypermethylated SBA, UCD, or TCD, respectively) appear further from the centre of the plot. Genes further to the right indicate an increase in expression and genes further to the left indicate a decrease in expression relative to the baseline group. The horizontal blue line indicates 0.05 *p* value threshold.

Differential expression analysis between CeD‐SBA and S‐SBA was performed for each gene (Figure 3A) to highlight any differentially expressed gene that could characterize CeD carcinogenesis. As expected, the *MLH1* gene was significantly downregulated in CeD‐SBAs (*p* = 0.024) compared with S‐SBAs; in fact, the CeD‐SBA group was significantly enriched by MLH1‐negative and *MLH1* hypermethylated adenocarcinomas, as already reported by Vanoli *et al* [26]. As also expected, CD8A (*p* = 0.0221), CD3G (*p* = 0.0394), and DNMT1 (*p* = 0.0462) transcripts were significantly upregulated in CeD‐SBAs compared with S‐SBAs, as the CeD‐SBAs are characterized by a prominent T‐lymphocyte infiltrate [26]. When compared with hyper‐SBAs, hypo‐SBAs showed mild overexpression of DNMT3A (*p* = 0.0036) and TET3 (*p* = 0.0084) transcripts together with the downregulation of IL2 expression (*p* = 0.0309, Figure 3B). Surprisingly, no *ORF1* or *ORF2* transcripts were overexpressed in hypo‐SBAs.

To further investigate the CeD carcinogenesis, a differential expression analysis was conducted between CeD‐SBAs and UCD or TCD mucosae (Figure 3C,D). The volcano plot of Figure 3C shows that CeD‐SBAs shared an expression profile similar to that of UCD mucosae, with only *TET1* and *TET2* transcripts being significantly downregulated (*p* = 0.0081 and *p* = 0.0085, respectively) and *CD4* and *IL6* transcripts significantly upregulated (*p* = 0.033 and *p* = 0.0287, respectively) in CeD‐SBAs compared with UCD mucosae. On the contrary, when CeD‐SBAs were compared with TCD mucosae, the differences were more remarkable, showing 15 genes differentially expressed among the two groups (Figure 3D). In detail, the *TET2* transcript was significantly downregulated (*p* <0.0001) in CeD‐SBAs, in keeping with the presence of an abundant cytotoxic CD8^+^ T‐cell infiltrate, as the loss of *TET2* promotes CD8^+^ T‐cell memory differentiation [33, 34]. Moreover, an extended upregulation of genes involved in inflammation, immune response, and T‐cell activity, such as *INFG*, *IL18*, *INFA1*, *IL12B*, *IL2*, *IL21*, *TET3*, *IL23A*, *IL1B*, *DNMT3B*, *RB1*, *CD4*, *IL6*, and *DNMT1*, was significantly associated with CeD‐SBAs. This extended gene upregulation can be caused by the presence of a significantly lower level of methylation in the CeD‐SBA group with respect to the TCD group.

## Discussion

For the first time, we observed that CeD‐SBAs are characterized by a significant loss of DNA methylation in LINE‐1 in comparison to controls such as CrD‐SBAs and S‐SBAs.

Considering the biological, clinicopathological, and prognostic features of CeD‐SBA, this is an unexpected result. Indeed, global hypomethylation of LINE‐1 is a well‐established feature in human cancer, and recent evidence has associated it with somatically acquired retrotransposition events that strongly correlate with poor prognosis, *TP53* mutation, high copy number alterations, and low immune activity [35, 36]. A recent pan‐cancer analysis of 2,954 whole genomes revealed that LINE‐1 insertion is the first most common type of somatic chromosomal rearrangement, including duplications, inversions, and translocations in various types of cancers [16]. Nevertheless, CeD‐SBAs are associated with a better prognosis among resected SBAs, and most of them are characterized by *MLH1* methylation and MSI rather than chromosomal instability [26]. Moreover, other genes besides *MLH1* have been demonstrated to be silenced in CeD‐SBA, such as *APC*, suggesting that aberrant CpG island methylation may be a critical step in CeD‐associated carcinogenesis [37]. We found LINE‐1 hypomethylation coupled with *MLH1* methylation in a significant fraction (34%, 13 out of 38) of CeD‐SBAs. These findings are different to what is observed in colorectal carcinomas, where there is evidence that LINE‐1 hypomethylation and MMR defects are two mutually exclusive markers [38] and that only in advanced stages may they be observed together [29]. Until now, the biological explanation for this remarkably high frequency of *MLH1* methylation in CeD‐SBA has not been known, and this new finding of aberrant CpG island methylation co‐existing with loss of LINE‐1 methylation in a fraction of CeD‐SBAs may provide important insight into the epigenetic mechanisms involved in the tumour initiation of these and other cancers associated with inflammation. These data led us to the working hypothesis that LINE‐1 hypomethylation may primarily be a hallmark of the immune response in CeD intestinal mucosa as in other autoimmune diseases, anticipating but not driving the onset of most of the CeD‐SBAs. Our findings obtained in non‐neoplastic duodenal samples fit with this hypothesis, demonstrating that the methylation levels of LINE‐1 repeats were not significantly different between CeD‐SBAs and mucosae from UCD patients. In addition, the restoration of normal LINE‐1 methylation levels in TCD mucosae strongly reinforces the idea that loss of DNA methylation is an intrinsic epigenetic feature of CeD, accompanying the immune response as a reversible mechanism in patients following a strict GFD. Also considering the expression analysis of enzymes modulating DNA methylation and inflammatory genes, we could observe that CeD‐SBAs shared an expression profile very similar to that of UCD mucosae showing a strong upregulation of genes involved in inflammation, immune response, and T‐cell activity compared with TCD mucosae. In this regard, LINE‐1 hypomethylation can also be considered as a sign of widespread epigenetic alterations, leading to specific changes of chromatin accessibility and upregulation of genes involved in the immune response (i.e. *IL6*) as well as of key genes involved in the functional organization of the genome. In particular, the role of *DNMT* and *TET* genes in both immune cell regulation and cancer has been extensively investigated, showing that the selectivity and specificity of substrates such as the DNA sequence may be unique to each DNMT and TET family member [33, 34, 39].

Another intriguing result of our study is the observation that in CeD and CeD‐SBAs, LINE‐1 hypomethylation does not lead to the expression of ORF1 and ORF2, with the only exception being for one CeD‐SBA. The human genome has encoded multiple defence strategies to silence the expression and mobility of transposable elements (TEs) in germ cells and normal tissues, including epigenetic repression by DNA methylation [40, 41] and many anti‐retrotransposon restriction mechanisms that are also known as anti‐retroviral factors [42, 43]. On the other hand, the role of TEs in promoting adaptability because of external changes such as biotic [44, 45] and abiotic factors including heat shock, DNA damage, UV radiation, climate, and chemical compounds is well established [3, 46, 47, 48, 49, 50, 51, 52, 53]. Recent impactful studies demonstrated that DNA‐demethylating agents upregulate immune signalling through the viral defence pathway that triggers an innate immune response [54, 55]. One mechanism is the generation of cytosolic nucleic acids by TEs that are recognized by sensors which in turn induce the production of IFN‐I such as IFNα and IFNβ, a pathway termed ‘viral mimicry’, as these sensors are usually activated by invading viruses [43, 56]. Although the IFN‐I response is vital for host protection against pathogens, aberrant chronic and/or episodic activation of IFN‐I is known as a hallmark in many autoimmune diseases showing hypomethylated and highly expressed LINE‐1 [17, 18, 19]. For this reason, while the main culprit behind chronic inflammation in autoimmune diseases was historically thought to be viruses, recent evidence suggests endogenous elements, including retrotransposons, as the prime suspects for the cause of persistent inflammation [57]. However, our results suggest that in CeD and CeD‐SBAs, hypomethylation is not sufficient to trigger retrotransposition, in contrast to other models of autoimmune diseases. This finding is in line with recent data reported by Jung *et al* [36], who found less LINE‐1 retrotransposition in tumours with high immune activity triggered by exogenous (e.g. Epstein–Barr virus infection in gastric cancer) or endogenous immunogens (e.g. MSI in gastric and colorectal cancers). These tumours seem to be protected against retrotransposition, in contrast to cancers showing low immune activity that are more prone to extensive LINE‐1 retrotransposition. Given the role that LINE‐1 hypomethylation may play in retrotransposition in many autoimmune diseases and in cancers, this work underscores the importance of a systematic immunohistochemical and mRNA expression analysis of ORF1 and ORF2 in CeD mucosae and in CeD‐associated cancers to better investigate the possible role of LINE‐1 retrotransposition in specific subsets of patients. Whilst more investigations are warranted to precisely highlight differentially methylated regions using epigenomic profiling approaches, the data provided in this study allow for a hypothesis that LINE‐1 retrotransposition may be under control during the immune response to gluten proteins in CeD patients, but at the same time LINE‐1 hypomethylation occurs and may have a crucial role in promoting cell adaptability during the gliadin‐related inflammatory process.

An interesting result obtained in our work is that CeD‐SBAs compared with TCD mucosae show a significant upregulation of crucial genes associated with long‐term inflammation or refractory CeD such as *IL6* [58, 59], *IL2* [60], *IL1* [61], *IL18* [62], *IFNA1*, *IFNG* [63], and *IL21* [22].

It is widely accepted that the risk of SBA development in CeD patients is significantly higher than in the general population, as supported by a meta‐analysis including 17 studies showing a pooled odds ratio of 14.4 [64]. Further, in a more recent nationwide Swedish study (excluding the first year after a CeD diagnosis to reduce detection bias), Emilsson *et al* reported a 3.05‐fold increased risk (95% CI 1.86–4.99) of SBA and a 5.73‐fold increased risk of small bowel adenoma (95% CI 3.70–8.88) in CeD individuals compared with age‐ and sex‐matched reference individuals [65]; additionally, the risk of SBA in CeD individuals was found to be highest during the first 10 years, while the median time between CeD and CeD‐SBA diagnosis was 2.7 years, a time‐lapse similar (2 years) to that observed in our cohort. Indeed, although the casual co‐occurrence of CeD and SBA cannot be excluded in a few CeD‐SBA cases, the distinctive molecular features of CeD‐SBA, including the significantly higher rates of dMMR/MSI, *MLH1* and *APC* methylation, and nuclear expression of β‐catenin compared with sporadic cases, support the role of CeD in the pathogenesis of CeD‐SBAs [26, 66, 67]. The results of our study further strengthen this association, as the rate of LINE‐1 hypomethylation was similar between active CeD mucosae and CeD‐SBAs but significantly higher than that of sporadic or CrD‐SBAs. In patients with resected SBAs, LINE‐1 methylation lacked a prognostic impact. Nevertheless, from a clinical point of view, the observation that LINE‐1 hypomethylation constantly accompanies the inflammatory and villous blunting processes in CeD indicates that this assay could be a helpful support to the histological evaluation of CeD follow‐up biopsies, where the distinction between mucosal healing and persistent villous blunting is clinically relevant [68], especially in cases of poorly oriented duodenal samples. Although more work is needed to support these findings, our data suggest that LINE‐1 hypomethylation should be investigated as a putative, reproducible surrogate biomarker of persistent villous blunting and/or refractory CeD in follow‐up duodenal biopsies. In conclusion, this works expands our knowledge about this complex immune‐mediated disease, suggesting for the first time the possible role of LINE‐1 hypomethylation in promoting cell adaptability during the gliadin‐related inflammatory process.

## Author contributions statement

AV and DF conceived and designed the experiments. AV, GA, MVL, PIB, GRC and ADS contributed to tumour material supply and collection of clinical information. LL and CG performed the experiments. LL, AV, NS, MA and DF analysed data and interpreted the results. LL and MA carried out bioinformatic analyses. LL, AV, PIB and DF prepared the manuscript, with contributions from all the other authors. MVL, SLR and ADS supervised the project. All the authors had final approval of the submitted and published versions.

## Supporting information


**Figure S1.** (A) Survival analysis comparing LINE‐1 hypomethylated SBA (hypo SBA, in red) to LINE‐1 hypermethylated SBA (hyper SBA, in black). (B) Survival analysis considering LINE‐1 methylation in CeD‐SBA (red), CrD‐SBA (green), and S‐SBA (blue)
**Figure S2.** NanoString® *MLH1* gene expression compared with MLH1 protein expression by immunohistochemistry in SBAs
**Figure S3.** Heatmap of the normalized data, scaled to give all genes equal variance, generated via unsupervised clustering showing an outlier case (case 87), and principal component analysis mapping high‐dimensional datasets onto a smaller number of highly informative dimensions showing an outlier case (case 87)

**Table S1.** NanoString® target genes included in the custom expression panel
**Table S2.** Comprehensive results from NanoString® expression analysis

## Data Availability

The data that support the findings of this study are available in the supplementary material of this article.
