# Supplementary material for: LINE‐1 hypomethylation characterizes the inflammatory response in coeliac disease associated‐intestinal mucosa and small bowel adenocarcinomas
Source: J Pathol. 2024 Nov 27;265(1):99–109. doi: 10.1002/path.6371 (PMC11638666; doi:10.1002/path.6371)
Supplement: Supplementary file 1 — Figure S1. (A) Survival analysis comparing LINE‐1 hypomethylated SBA (hypo SBA, in red) to LINE‐1 hypermethylated SBA (hyper SBA, in black). (B) Survival analysis considering LINE‐1 methylation in CeD‐SBA (red), CrD‐SBA (green), and S‐SBA (blue) Figure S2. NanoString® MLH1 gene expression compared with MLH1 protein expression by immunohistochemistry in SBAs Figure S3. Heatmap of the normalized data, scaled to give all genes equal variance, generated via unsupervised clustering showing an outlier case (case 87), and principal component analysis mapping high‐dimensional datasets onto a smaller number of highly informative dimensions showing an outlier case (case 87) [file PATH-265-99-s001.docx]

**LINE-1 hypomethylation characterizes the inflammatory response in coeliac disease associated-intestinal mucosa and** **small bowel adenocarcinomas**

L Libera *et al. J Pathol* <https://doi.org/10.1002/path.6371>

**Supplementary Figures S1–S3**

**Supplementary Tables S1 and S2 are provided as a separate Excel file**


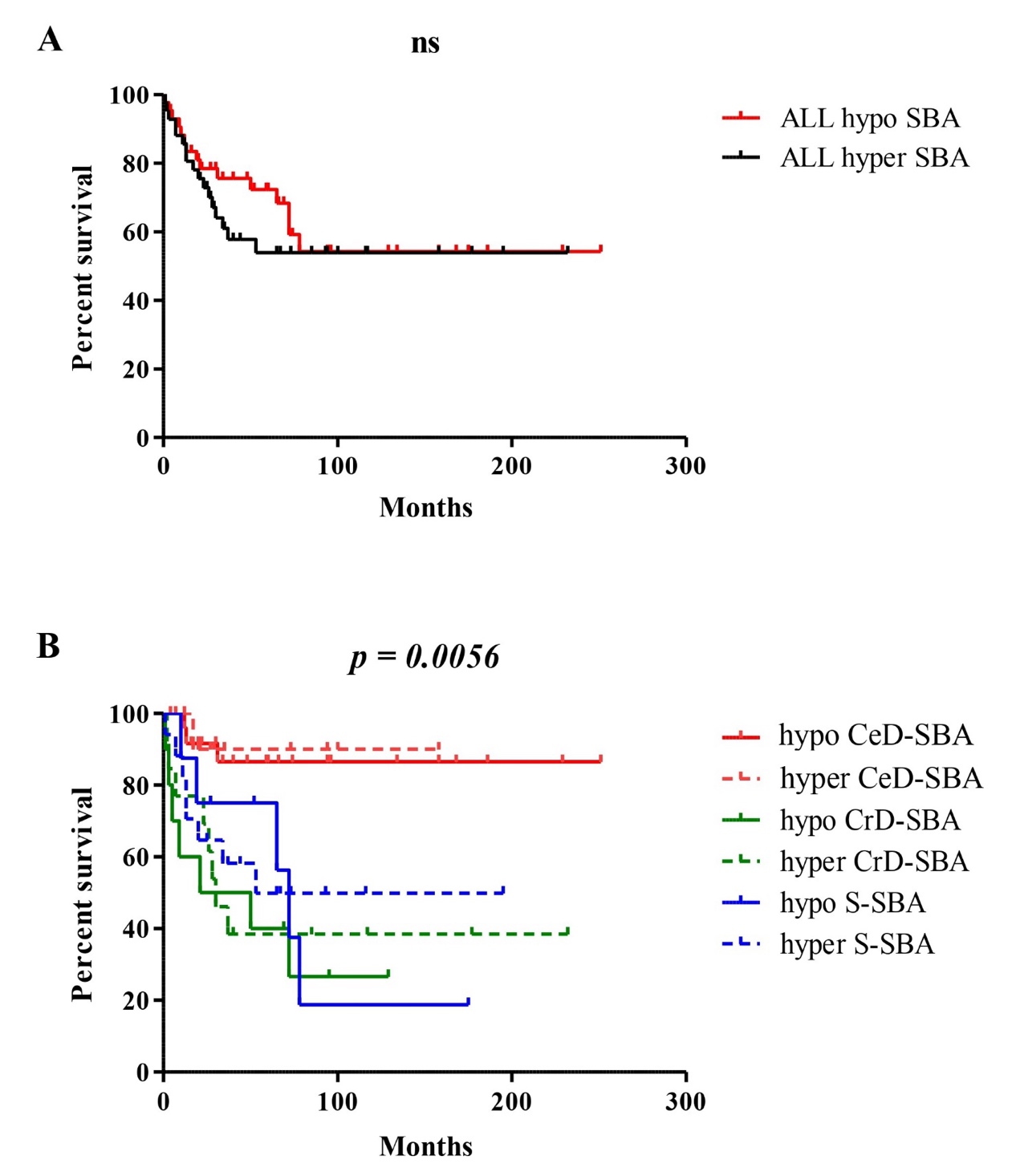


**Figure S1. (A) Survival analysis comparing LINE-1 hypomethylated SBA (hypo SBA, in red) with LINE-1 hypermethylated SBA (hyper SBA, in black). (B) Survival analysis considering LINE-1 methylation in CeD-SBA (red), CrD-SBA (green), and S-SBA (blue).**


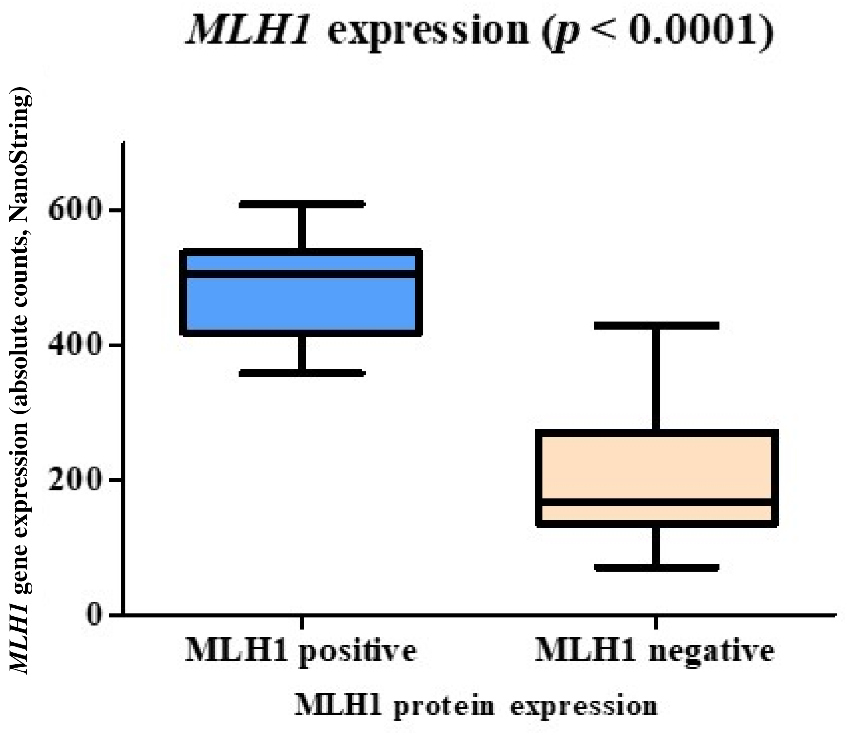


**Figure S2. NanoString^®^ *MLH1* gene expression compared with MLH1 protein expression by immunohistochemistry in SBAs.** The *t*-test analysis revealed a significant correlation between NanoString^®^ *MLH1* absolute counts and MLH1 protein expression (*p* < 0.0001).


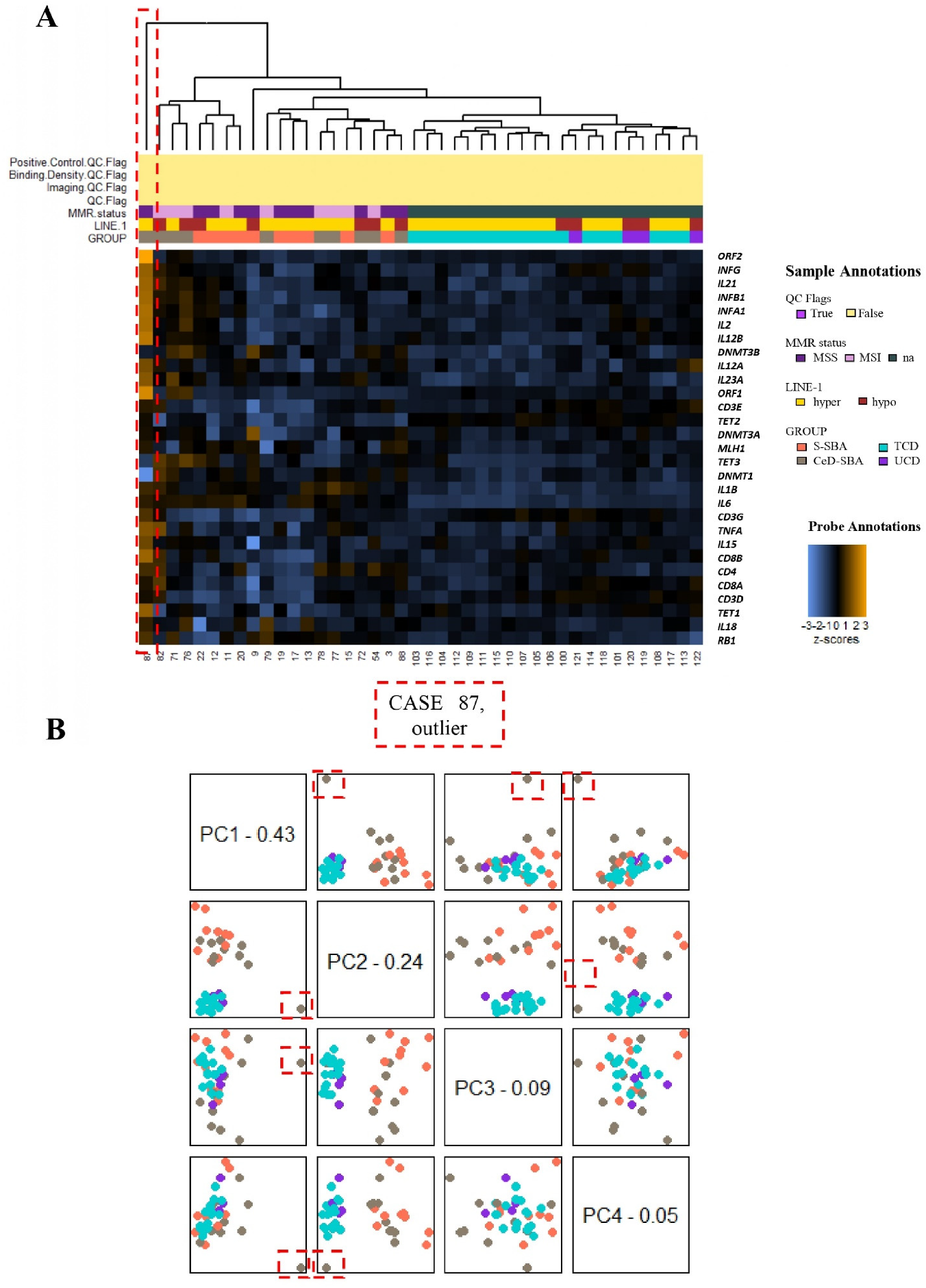


**Figure S3. Heatmap of the normalized data, scaled to give all genes equal variance, generated via unsupervised clustering showing an outlier case (case 87), and principal component analysis mapping high-dimensional datasets onto a smaller number of highly informative dimensions showing an outlier case (case 87).** (A) Expression values are scaled by gene to have a mean of 0 and a standard deviation (SD) of 1 and then truncated at ± 3 SDs to preserve greater clarity in colour change within the largest proportion of data (99% of the data should fall within ± 3 SDs of the mean). Sample annotations are listed at the top of the heatmap: MMR status (MSS in violet; MSI in pink; not available in green); LINE-1 status (hypermethylated in yellow; hypomethylated in red); class groups (S-SBA in orange; CeD-SBA in grey; TCD in light blue; UCD in violet). The genes are displayed in rows. Each column is a unique sample, with a sample label displayed below the heatmap. The outlier sample, case 87 is marked with a red dashed square. MMR, mismatch repair status; MSI, microsatellite unstable; MSS, microsatellite stable, na, not available; hyper, LINE-1 hypermethylated; hypo, LINE-1 hypomethylated; S-SBA, sporadic SBA; CeD-SBA, coeliac disease SBA; TCD, mucosae from coeliac disease patients in treatment; UCD, mucosae from coeliac disease untreated patients. (B) Principal component analysis maps high-dimensional datasets onto a smaller number of highly informative dimensions showing an outlier case (case 87). The figure shows the first four principal components of the gene expression data plotted against each other and coloured by class groups (S-SBA in orange; CeD-SBA in grey; TCD in light blue; UCD in violet). This plot shows the clustering of TCD and UCD mucosae separated from SBA samples. The outlier sample, case 87, is marked with a red dashed square.
